# Psychometric properties of Addenbrooke’s Cognitive Examination III (ACE-III): An item response theory approach

**DOI:** 10.1371/journal.pone.0251137

**Published:** 2021-05-06

**Authors:** Carlos Calderón, Christian Beyle, Oscar Véliz-García, Juan Bekios-Calfa

**Affiliations:** 1 Escuela de Psicología, Universidad Católica del Norte, Antofagasta, Chile; 2 Departamento de Psicología, Universidad Católica de Temuco, Temuco, Chile; 3 Departamento de Ingeniería en Sistemas y Ciencias de la Computación, Universidad Católica del Norte, Antofagasta, Chile; University of Florence, ITALY

## Abstract

The Addenbrooke’s Cognitive Examination III is one of the most widely used tests to assess cognitive impairment. Although previous studies have shown adequate levels of diagnostic utility to detect severe impairment, it has not shown sensitivity to detect mild decline. The aim of this study was to evaluate the psychometric properties of Addenbrooke’s Cognitive Examination III in a large sample of elderly people through Item Response Theory, due to the lack of studies using this approach. A cross-sectional study was conducted with 1164 people from the age of 60 upwards, of which 63 had a prior diagnosis of Alzheimer dementia. The results showed that, globally, the Addenbrooke’s Cognitive Examination III possesses adequate psychometrics properties. Furthermore, the information function test shows that the subscales have different sensitivity to different levels of impairment. These results can contribute to determining patterns of cognitive deterioration for the adequate detection of different levels of dementia. An optimized version is suggested which may be an economic alternative in the applied field.

## Introduction

Dementias are one of the main causes of malfunction and dependency in adulthood [1]. According to the World Health Organization [2], in 2015 50 million people suffered from some kind of dementia, and the number is expected to triple by 2030. This neurocognitive impairment affects not only those who suffer from it but also their caregivers, family members, the community, and general society.

Previous evidence has presented the benefits of early detection and treatment of dementia. These benefits are not only of a clinical nature [3] but also economic [4–6], social [7], and in terms of public health [8,9], both for the patient and their relatives and caregivers. Therefore, it is crucial that professionals, both specialists and non-specialists, have access to accurate, time-saving, and easy-to-use screening tools that allow early detection of dementia.

The Classical Test Theory (CTT) traditionally used for psychometric analysis of cognitive tests, uses the sum of raw scores both for diagnosis and in order to estimate the level of cognitive decline in people. This method is easy to apply and is based on the assumption that all items reflect a common trait or ability from which the level of impairment can be inferred [10].

However, that assumption can be seen as unrealistic, since it assumes that all items have the same precision and difficulty. The direct sum of the raw scores does not consider the differences in the metric properties of the items, and the information provided by the different response patterns remains unknown. In other words, two people with the same number of correct responses could have different levels and/or paths of decline depending on the specific items they have hit.

To overcome these and other limitations of CTT, the Item Response Theory (IRT) was presented several years ago [11–16]. Unlike the CTT—which associates the item’s response to the total score—the IRT relates the probability of success of each item with the estimation of the latent trait through a logistic function called Item Characteristic Curve (ICC). There are different IRT models depending on the number of parameters that are estimated to define the ICC. The most widely used is the 2-parameter logistic model. This ICC model is defined by two parameters: difficulty (b-parameter) and discrimination (a-parameter). These parameters provide useful information about technical properties of the ICC.

The b-parameter corresponds to the level of the latent trait at which the probability of success is 50% (p = .5). On the other hand, a-parameter informs on the slope of the curve at the point of the trait continuum where the b-parameter is located. It informs about how well the item manages to differentiate between subjects on a particular area of the trait.

The overall performance of the test can be evaluated through the information function. It shows the test’s acuteness/discernment capability, which can be estimated for each level of the latent trait. Therefore, IRT provides information for determining deterioration sequences/paths and at the same time increasing the test’s sensitivity to certain impairment levels [17–20]. Furthermore, IRT provides global and item related goodness-of-fit indices, allowing an adequate evaluation of the measures.

In order to establish a hierarchy of items and scales of difficulty it could be helpful to determine the sequence of deterioration or the level of dementia severity based on the patient’s answers. Following different impairment trajectories could help to diagnose different types of dementia [19]. Finally, knowing the discrimination capability of the items allows to examine their sensitivity at certain levels of impairment.

Despite the mentioned advantages, there is little prior evidence of the psychometric properties of tests that evaluate cognitive impairment using IRT’s framework. Existing research has evaluated a small number of tests [10,18–24], or have used non-parametric IRT models [25–31].

Cognitive screening for dementia faces many difficulties, especially in early stages of impairment. Considering that cognitive performance change is a key symptom within the diagnostic criteria [32,33], it is necessary for tests to possess adequate sensitivity at different stages of cognitive deterioration in order to detect unusual patterns in cognitive performance. A competent cognitive assessment must have the capability to distinguish between age-related decline and dementia-related impairment, and differentiating mild from more severe stages of the disease [33]. Additionally, it should help to define a cognitive performance profile that provides information on the different cognitive domains, allowing the detection of different paths of deterioration for diagnosis and treatment [34].

There is currently a large number of cognitive assessment tests to screen for dementia. Among these, the Mini-Mental State Examination [35] is the most widely used. Despite this, several studies report its limitations as a screening test, among which the following can be found: it presents low sensitivity to detect dementia in the initial stages [36,37], shows little diagnostic utility in primary care [36], presents difficulties in detecting dementias other than Alzheimer’s [37], it has imprecise cut-off scores that lead to inaccurate diagnostic classifications [38] and has limited evidence of generalization validity, being affected by sociocultural elements [39].

One of the tests that attempts to overcome these limitations is the Addenbrooke’s Cognitive Examination (ACE) [40]. The third version of the test (ACE-III) has shown adequate diagnostic effectiveness for detecting Alzheimer’s and other types of dementia [41,42]. It provides information from five cognitive domains (attention, memory, language, verbal fluency, and visuospatial abilities), which makes it an excellent alternative to obtain a deterioration profile [43]. Despite several studies evaluating the validity of ACE-III and its adaptation to a large number of languages and cultural contexts [44], most of these studies focus on analyzing its diagnostic utility and its relation to other tests or variables [45–54]. At present, few studies have conducted a psychometric analysis of ACE-III from parametric IRT approaches [55].

Therefore, the aim of this study is to perform a psychometric evaluation of the ACE-III from a 2-parameter IRT model. The results of this research will provide evidence about the metric properties of the test. The results of the estimation of the IRT model could help to determine levels of impairment progression, to determine the most sensitive items and subscales to certain levels of impairment, and to develop profiles of progression.

## Materials and methods

The study was approved by the Scientific Ethics Committee of the Universidad Católica del Norte, under the resolution 004/2018. Informed consent and/or consent was obtained in writing from all research participants.

### Participants

A cross-sectional study was conducted with 1164 people from the age of 60 upwards. Writing and reading were evaluated. Regarding sex, 288 (26.2%) participants were men and 810 were women (73.8%). The mean age was 71.8 years (SD = 7.9) and the mean years of schooling was 10.9 (corresponding approximately to complete secondary education). 63 participants had a prior diagnosis and treatment of Alzheimer dementia, confirmed in secondary specialty care (geriatrics, neurology, and/or psychiatry) that was periodically registered in their medical history. The diagnosis was established by means of scores equal to or greater than 4 points in the Global Deterioration Scale (GDS). The time of evolution of the disease was not considered due to differences in the time of diagnosis and the course of the disease. The rest of the participants did not present a medical diagnosis that could affect cognitive functioning. Exclusion criteria considered the absence of severe sensory disturbances (without correction), consciousness, and other medical conditions different from dementia that could affect cognitive functioning. A suspension evaluation criterion was considered in three cases in which comprehension of the task’s instructions was affected (different from alteration of consciousness).

### Measures

The ACE-III is a hetero-applied test for optimal performance. The Chilean version of the test was used [53] which consists of 81 items and is composed of five subscales: orientation-attention (18 items and 18 points), memory (25 items and 26 points), verbal fluencies (2 items and 14 points), language (27 items and 26 points), and visuospatial skills (11 items and 16 points), with 100 being the maximum total score. According to its latest report [53], the test presents an α-Cronbach = 0.87, establishing 86 points (98.5% sensitivity and 82% specificity) as a cut-off score for dementia detection.

### Procedures

Recruitment was carried out in the primary healthcare network (PHC) in social groups of older people, and through a public notice through the National Service for the Elderly of Chile. The evaluations were carried out by a team of three psychologists and a neuropsychologist with training in cognitive evaluation.

### Data analysis

To contrast the assumptions of unidimensionality and local independence necessary for the adequate estimation of the IRT models, the total scale and the subscales have been analyzed using Confirmatory Factor Analysis (CFA). In all analyses we have specified a one-factor model. To estimate these models, we have used the Weighted Last Square with mean and variance adjusted (WLSMV) estimation method available in the Mplus software (v.8) which is recommended for dichotomous variables. Once the unidimensionality of the data had been evaluated, the estimation of the 2-parameter IRT models was carried out with the Marginal maximum likelihood estimation method and the MHRM algorithm was used. Finally, for the goodness-of-fit evaluation of the models, we have used the fit indices commonly used in research practice. For the global fit, we have used the M_2_ statistic and the derived CFI, TLI and RMSEA indices. In the case of the local fit of the items, we have used the *S*−*χ*^2^ and RMSEA statistics. All analyses were carried out on the total sample of participants.

## Results

As previously mentioned, the study attempted to evaluate the psychometric properties of the ACE-III test from an IRT framework. The results are presented in three sections. First, the results of the goodness-of-fit of each subscale to a one-factor model through Confirmatory Factor Analysis (CFA). The goodness-of-fit to data to a one-factor model allows us to check the assumptions of unidimensionality and local independence, which are needed for the proper estimation of IRT models. Second, the results of the estimation of parameters for the IRT models, together with the goodness-of-fit indices of the items, and the global fit indices of the subscales. Additionally, a revised version for each subscale is presented, which have been obtained by selecting the items that presented an adequate fit to the IRT model.

### Confirmatory factor analysis

As mentioned above, we have evaluated the unidimensionality assumption through a CFA for each subscale. The Weighted Least Square with mean and variance adjusted estimation method (WLSMV) was used for the estimation of the models, which is appropriate for categorical variables, using the statistical software Mplus v.8. In order to evaluate the parameters fit, in addition to the *χ*^2^ statistic, the most used goodness-of-fit indices in psychometry (RMSEA, TLI and CFI) were obtained. The results are presented in Table 1.

**Table 1:** Goodness of fit indices of each subscale to the unidimensional model (AFC).

	χ^2^	df	p	RMSEA	RMSEA (IC90%)	TLI	CFI
**Orientation**	76.681	35	.000	.032	.022 - .042	.994	.995
**Attention**	109.227	20	.000	.062	.051 - .074	.977	.983
**Memory**	969.592	104	.000	.085	.080 - .090	.950	.956
**Language**	824.565	230	.000	.048	.044 - .051	.957	.961
**Viso-construction**	239.134	65	.000	.048	.042 - .055	.979	.982

Note: χ^2^ = Chi-square; df = degrees of freedom; p = p-value; RMSEA = Root mean square error of approximation.

The results of Orientation and Attention subscales are presented separately, because a two-factor model shows a significant improvement in the fit compared to the one-factor model. These findings have been presented in previous studies [51]. Goodness-of-fit indices show excellent fit to all subscales. Only one of the indices in the Memory subscale shows a poor fit (RMSEA = .085). Despite this, the results allow us to assume the unidimensionality of the subscales, since previous studies have shown that IRT models are robust enough to non-excessive violations of this assumption [56,57].

### Item response theory

To assess each subscale from the IRT framework, a logistic model of 2-parameters was adopted. The Marginal maximum likelihood (ML) estimation method [58], available in the Mirt package for R software [59]. Item fit was assessed obtaining the *S*−*χ*^2^ statistic, which tests the hypothesis of equality between the model’s predicted response probabilities and the observed response frequencies. Probabilities associated with *S*−*χ*^2^ higher than .05 indicated an adequate fit of the item parameters. Additionally, the RMSEA related to *S*−*χ*^2^ was obtained in order to evaluate the magnitude of lack of fit. To obtain a revised version, items with lack of fit were deleted. For both the original and the revised version, we present the global goodness-of-fit statistics based on the M_2_ [60]. The results of the revised version are presented below. the reader can consult the supplements for the results of the full versions of the subscales.

#### a) Orientation subscale

The *S*−*χ*^2^ statistic shows that “date” and “commune”(In Chile, the smallest administrative division governed by a mayor assisted by a municipal council) (see S1 Table) present a significant lack of fit (*p*<.05). In this regard, it might be suggested that failure in both items could be more related to educational and occupational differences than to cognitive impairment (see discussion). Based on the above, these items have been removed from the revised version. The Table 2 shows the estimated parameters for the proposed Orientation subscale.

**Table 2:** Parameters estimated and item fit indices of proposed version of orientation dimension.

	Parameters estimated				Items fit indices			
	a	S.E.	b	S.E.	S-χ^2^	df	p	RMSEA
**Day**	2.526	.269	-1.499	.085	3.308	4	.508	.000
**Month**	4.848	.704	-1.308	.061	1.877	3	.598	.000
**Year**	3.936	.538	-1.004	.056	2.329	3	.507	.000
**Season**	2.393	.248	-1.225	.074	2.349	4	.672	.000
**Country**	2.860	.450	-2.359	.153	7.386	5	.193	.021
**City**	4.350	.770	-1.980	.099	2.819	3	.420	.000
**Street**	3.520	.440	-1.614	.081	5.284	4	.259	.017
**Number**	1.947	.212	-1.786	.117	3.289	5	.655	.000

Note: a = a-parameter; S.E. = Standard error; b = b-parameter; S-χ^2^ = Goodness of fit index S-χ^2^; df = degrees of freedom; p = p-value; RMSEA = Root mean square error of approximation.

The values of a*-*parameter*s* show these items with a high discrimination, mostly over 2. The b-parameters indicate the highest discrimination of the items between -2.5 and -1.0 of the trait level (θ). Thereby, the Orientation subscale reaches its maximum discrimination within the 25% of people with lower performance, which is expected in a test that evaluates cognitive impairment.

As observed, values for this revised version are very similar to the original. All items present statistical fit to the model (*p*>.05).

Table 3 displays the fit indices of all the subscales to the IRT model. The first two rows show the fit of the original version and the proposed revised version of the Orientation subscale. Although both models present good fit indicators, the revised version shows a significant fit improvement (*Δχ*^2^ = 1838.979; *p*<.05).

**Table 3:** Goodness of fit indices of the 2 parameter IRT model of each original subscale and reduced proposed version.

		M2	df	p	RMSEA	RMSEA (IC90%)	SRMR	TLI	CFI
**Orientation**	**Original**	65.169	35	.001	.028	.017 - .038	.055	.995	.996
	**Reduced**	32.221	20	.041	.023	.005 - .038	.049	.997	.998
**Attention**		261.935	20	.000	.104	.093 - .115	.167	.927	.948
**Memory**	**Original**	1478.733	299	.000	.059	.056 - .062	.051	.970	.972
	**Reduced**	654.341	104	.000	.067	.062 - .072	.049	.968	.973
**Language**	**Original**	1138.443	299	.000	.050	.047 - .053	.058	.977	.979
	**Reduced**	693.999	189	.000	.048	.044 - .052	.055	.980	.982
**Viso-construction**	**Global**	1290.242	104	.000	.101	.095 - .106	.107	.924	.935
	**V. Recognition**	1021.378	20	.000	.211	.200 - .124	.124	.772	.837
	**Construction**	38.948	20	.007	.029	.015 - .043	.068	.996	.997

Note: M_2_ = M_2_ statistic; df = degrees of freedom; p = p-value; RMSEA = Root mean square error of approximation; SRMR = Standardized Root Mean-Square; TLI = Tucker–Lewis index; CFI = Comparative Fit Index.

#### b) Attention subscale

Similarly as the Orientation subscale, the Attention subscale items present high levels of discrimination and difficulty parameters between -2.5 and 0 (see Table 4). Further, the *S*−*χ*^2^ statistic identifies three items (*key*, *rest1*, and *rest4*) with lack of fit to data. Removing these items would produce the subscale to contain an insufficient number of items for an adequate estimation of the person parameter. Additionally, two of these correspond to items of progressive presentation (*rest1* and *rest 4*: progressive subtraction), which would advise against their deletion. Finally, the global fit indices show a clear lack of goodness-of-fit (RMSEA>.08; CFI < .95; TLI < .95). Overall, the Attention subscale shows no evidence of global goodness-of-fit.

**Table 4:** Parameters estimated and item fit indices of attention dimension.

	**Parameters estimated**				**Items fit indices**			
	a	S.E.	b	S.E.	S-χ^2^	df	p	RMSEA
**Lemon**	2.854	.493	-2.566	.183	1.033	1	.309	.005
**Key**	2.315	.366	-2.636	.208	17.250	2	.000	.082
**Door**	2.831	.477	-2.515	.178	1.811	1	.178	.027
**Rest 1**	2.436	.243	-1.081	.067	13.682	3	.003	.056
**Rest 2**	2.126	.175	.220	.049	4.029	2	.133	.030
**Rest 3**	3.119	.286	-.030	.043	0.861	2	.650	.000
**Rest 4**	3.887	.426	.106	.041	6.297	2	.043	.044
**Rest 5**	3.154	.294	.101	.043	2.494	2	.287	.015

Note: a = a-parameter; S.E. = Standard error; b = b-parameter; S-χ^2^ = Goodness of fit index S-χ^2^; df = degrees of freedom; p = p-value; RMSEA = Root mean square error of approximation.

#### c) Memory subscale

The goodness-of-fit indices shows that 5 out of 26 items of Memory subscale present lack of fit (see S2 Table). Assuming that the items Declarative memory, Episodic free recovery, and Episodic recognition are related to genuine memory deficits [61], they represent stronger evidence of the specific memory processes in the context of dementia screening [61–63], we evaluated a revised version of the scale deleting the Working memory item and not scoring Episodic encoding items (Table 5).

**Table 5:** Parameters estimated and item fit indices of proposed version of memory dimension.

		**Parameters estimated**				**Items fit indices**			
		a	S.E.	b	S.E.	S-χ^2^	df	p	RMSEA
**Declarative memory**	**Actual president**	2.446	.213	-1.392	.076	17.954	11	.083	.024
	**Military government**	2.514	.225	-1.442	.077	8.399	10	.590	.000
	**USA president**	1.498	.115	-.328	.059	12.718	13	.470	.000
	**Murdered USA pres.**	1.952	.150	-.877	.064	12.883	13	.457	.000
**Episodic Free recovery**	**Miguel (2)**	1.649	.129	.285	.054	5.929	12	.920	.000
	**González (2)**	2.392	.182	-.010	.045	7.822	11	.729	.000
	**Avenida (2)**	2.077	.157	.055	.048	10.531	11	.483	.000
	**Imperial (2)**	2.109	.169	.483	.051	8.817	10	.550	.000
	**68 (2)**	2.937	.236	.006	.042	7.124	10	.714	.000
	**Caldera (2)**	2.458	.191	.062	.045	9.504	11	.575	.000
	**Copiapó (2)**	3.117	.248	-.440	.045	18.820	11	.064	.025
**Episodic Recognition**	**Miguel González (3)**	2.071	.158	-.854	.061	12.966	13	.450	.000
	**Avenida Imperial (3)**	1.954	.151	-.950	.066	10.863	13	.622	.000
	**68 (3)**	1.981	.152	-.871	.063	24.861	13	.024	.028
	**Caldera (3)**	2.517	.196	-.815	.056	15.653	12	.208	.016
	**Copiapó (3)**	3.407	.337	-.128	.064	7.686	9	.566	.000

Note: a = a-parameter; S.E. = Standard error; b = b-parameter; S-χ^2^ = Goodness of fit index S-χ^2^; df = degrees of freedom; p = p-value; RMSEA = Root mean square error of approximation.

Table 5 shows the high discrimination of this subscale. Regarding their difficulty, the items Episodic free recognition and Declarative memory reach values around -1, while the difficulty of Episodic free recovery are closer to the mean (θ = 0). The Table 5 show that, with the exception of one item (“*recognition 68”*), all the items show adequate fit. Although both versions of the subscale (original and revised) have adequate global goodness-of-fit, the revised version might be seen as a more parsimonious model.

Based on the values of the estimated b-parameters of the different tasks, the results show that items of the Episodic free recovery task have greater sensitivity in mild levels of impairment (*b* = ±0), while items in the tasks of Declarative memory and Episodic recognition have greater sensitivity at more severe levels of deterioration (*b* = −1). These findings support the possibility of establishing performance-based impairment levels among different memory tasks.

#### d) Language subscale

The estimates for the revised version of Language subscale are shown in Table 6. The results of the full version are presented in the supplements.

**Table 6:** Parameters estimated and item fit indices of proposed version of language dimension.

	**Parameters estimated**				**Items fit indices**			
	a	S.E.	b	S.E.	S-χ^2^	df	p	RMSEA
**Instruction 1**	1.996	.183	-1.682	.105	16.257	17	.506	.000
**Instruction 2**	2.151	.209	-1.963	.127	4.864	17	.998	.000
**Instruction 3**	1.275	.111	-1.238	.105	8.699	16	.925	.000
**Repeat phrase 1**	1.358	.120	-1.172	.091	12.884	15	.611	.000
**Repeat phrase 2**	1.815	.158	-1.440	.093	11.300	15	.731	.000
**Create phrase 1**	2.740	.240	-1.363	.075	10.039	14	.759	.000
**Create phrase 2**	1.955	.150	-0.608	.058	6.042	12	.914	.000
**Repeat words 2**	1.326	.125	-0.074	.055	11.590	12	.479	.000
**Spoon**	4.608	.921	-2.378	.130	2.722	1	.099	.039
**Book**	1.986	.200	-1.973	.126	23.614	17	.130	.018
**Penguin**	3.086	.189	-1.409	.069	17.452	12	.133	.020
**Anchor**	2.345	.210	-1.404	.079	11.820	15	.693	.000
**Camel**	2.362	.113	-1.473	.062	9.112	15	.872	.000
**Harp**	2.488	.123	-1.160	.117	9.710	13	.717	.000
**Barrel**	1.338	.368	-1.520	.072	19.805	17	.284	.012
**Crown**	3.674	.156	-1.475	.079	21.274	12	.050	.026
**Crocodile**	1.861	.172	-1.202	.104	17.121	15	.312	.011
**Monarchy**	2.878	.261	-1.425	.076	18.534	15	.236	.014
**Reptile**	3.067	.282	-1.501	.079	9.141	14	.822	.000
**Antarctica**	3.656	.335	-1.370	.069	7.532	12	.821	.000
**Nautical**	2.486	.205	-1.218	.071	21.025	13	.072	.023

Note: a = a-parameter; S.E. = Standard error; b = b-parameter; S-χ^2^ = Goodness of fit index S-χ^2^; df = degrees of freedom; p = p-value; RMSEA = Root mean square error of approximation.

Because three items scored 0, 1 or 2 points, they have been recoded into dummy variables (*repeat the sentence*, *write a sentence* and *repeat words*). The a-parameters show that all items have high discrimination, while b*-*parameters indicate maximum discrimination between trait levels from -2.5 to 0. Regarding the fit of the model, five items show lack of fit: “repeat words 1”, “kangaroo”, “rhino”, “accordion” and “repeat word 4” (see supplement). The lack of fit of “repeat words 1” suggests that conceding full score in case of success, and zero points in case of at least one failure, is more appropriate than using partial scores. For the rest of the items, a cultural/educational effect is likely to affect their fit (e.g. limited knowledge of the animals, or the accordion and bandoneon lookalike) and acquiescence effect might be found (a hippopotamus appears in a previous task, which is a common error for the rhino item). By deleting these items, the fit indices improve reaching an adequate goodness-of-fit, both to item and model levels (Table 3).

#### e) Viso-construction subscale

Table 3 shows the global lack of fit of the subscale (CFI>.95; TLI>.95; RMSEA < .06). As the items of the subscale involve two well-differentiated tasks (visual recognition and visual construction), we have specified a different model for both sets of items. The estimated parameters of both models are presented in the Table 7.

**Table 7:** Parameters estimated and item fit indices of proposed version of visual construction and visual recognition subscales.

	Parameters estimated				Items fit indices			
	a	S.E.	b	S.E.	S-χ^2^	df	p	RMSEA
**Visual Construction**	1.726	.142	-.588	.053	41.773	5	.000	.081
	1.983	.163	-.726	.053	28.963	5	.000	.065
	1.481	.124	-.037	.051	20.607	5	.001	.053
	3.026	.340	-1.631	.085	22.210	4	.000	.064
	57.119	12.106	-.590	.025	377.071	2	.000	.409
	49.758	47.637	-.434	.034	516.016	2	.000	.479
	2.856	.240	-.637	.040	24.696	5	.000	.059
	2.147	.174	-.095	.040	35.888	5	.000	.074
**Visual Recognize**	3.234	.462	-1.804	.102	3.060	4	.548	.000
	2.502	.321	-1.602	.100	11.502	4	.021	.041
	2.151	.274	-1.844	.126	2.493	4	.646	.000
	2.628	.349	-1.667	.103	4.991	4	.288	.015
	1.886	.236	-1.717	.126	4.543	4	.338	.011
	5.412	1.154	-1.796	.089	14.825	3	.002	.059
	5.251	1.104	-1.964	.100	11.292	4	.023	.040
	5.006	1.024	-2.025	.105	12.707	3	.005	.054

Note: a = a-parameter; S.E. = Standard error; b = b-parameter; S-χ^2^ = Goodness of fit index S-χ^2^; df = degrees of freedom; p = p-value; RMSEA = Root mean square error of approximation.

Two important results may be highlighted. First, all the Viso-construction items present a lack of fit to the model. In contrast, in the Visual Recognition task, four out of eight items show a lack of fit, but none of them present an excessive misfit (RMSEA < .06). Second, the global fit indices show that only Visual Recognition presents an adequate fit. These results suggest that a revised version of this subscale should comprise only Visual recognition items, in order to avoid the Viso-construction items’ lack of goodness-of-fit.

#### f) Information function

**Fig 1:**
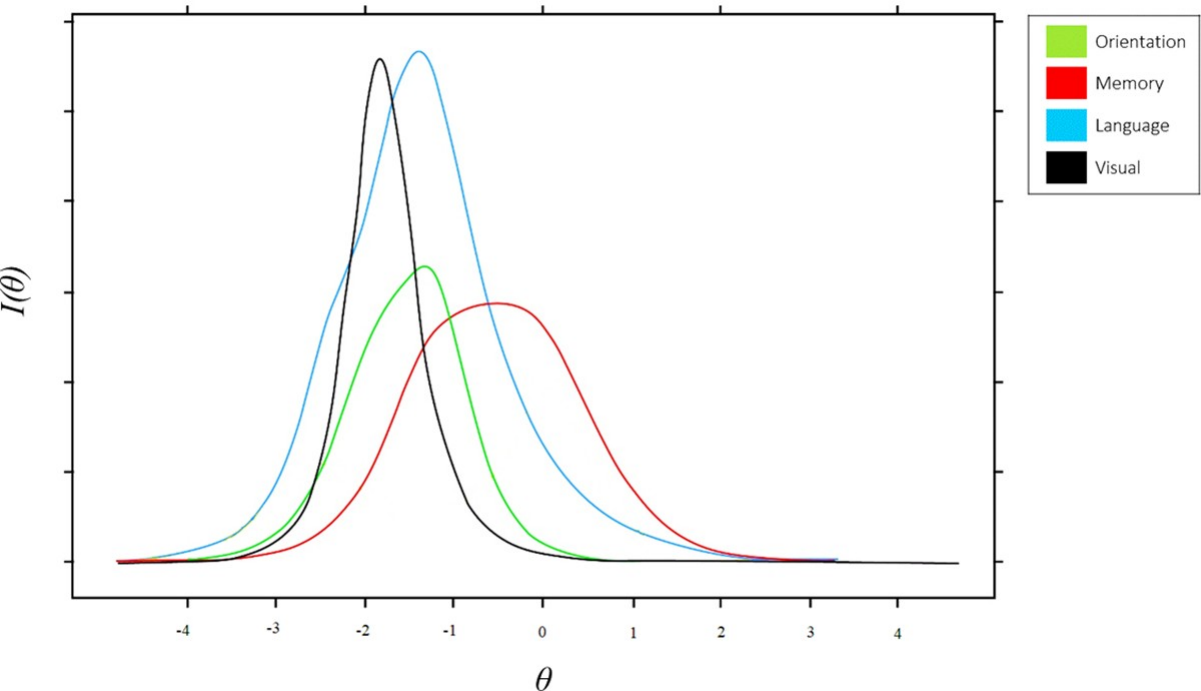
Information functions curves of subscale of ACE-III.

In order to determine the sensitivity of the ACE-III’s revised subscales for the different levels of cognitive decline, the information function of each subscale has been estimated. The information function corresponds to an indicator of the test’s level of precision (reliability) or discrimination capacity. Once the information function is calculated for each interval of the trait level, the information function curve is obtained. Fig 1 compares the information curves of the Orientation, Language, Memory and Visual recognition subscales.

Two findings may be highlighted. First, the Memory subscale presents higher levels of sensitivity at mild levels of the trait. In addition, it is the subscale that presents a greater curve amplitude compared to the rest of the subscales. This last result was mainly due to the differences in the difficulty parameters between the Episodic free recovery task and the Declarative memory and Episodic recognition tasks. These findings support the use of the memory subscale for the detection of mild cognitive impairment. Second, the location of the information curve peaks with respect to the continuum of trait level may be indicating a decline pattern, which begins with memory decline, then with language and orientation simultaneously, and finally ending with visual recognition decline.

## Discussion

The purpose of this study was to evaluate the psychometric properties of ACE-III from the framework of IRT in a large sample of older people. This objective was set due to two reasons. First, the lack of studies analyzing the psychometric properties of the measuring of cognitive impairment in general, in the ACE-III. Second, the advantages that IRT has over classic approaches. We were interested in the possibility of determining patterns and/or sequences of deterioration from the set of parameters of the subscales, contributing with evidence towards the development of indicators for the detection of mild cognitive deterioration, as well as the possibility of constructing profiles which can contribute towards the development of indicators for the differential diagnosis of different types of deterioration.

Concerning the overall results, three findings are noteworthy. First, the presence of two well-differentiated latent factors for the orientation/attention subscale. This result suggests that it would be best to obtain scores for the orientation tasks separately for an adequate evaluation of cognitive performance.

Additionally, the analysis of the attention tasks’ items shows a lack of model fit. From a theoretical point of view, it is likely that lack of model fit is related to the complexity of an adequate neuropsychological assessment of attention [64–66]. This subscale is mainly composed of subtraction-related items which involve arithmetic skills that depend on neural pathways other than those related to attention [67,68]. Additionally, the remaining three items (repeat lemon-key-door) is a very small number of items to estimate the subjects’ trait level adequately. Therefore, it is recommended that the subscale of attention in the ACE-III be used with caution until there are works that propose more adequate forms for assessing attention.

Second, in relation to the pattern of cognitive deterioration, the information curves by subscales show a clear pattern that begins with the deterioration of memory capacity (especially vis-à-vis the tasks of episodic free recovery) followed by the deterioration in orientation and language capacity and, finally, problems in visual recognition. Associated with these findings, the greater sensitivity shown by the memory subscale in the middle levels of the latent trait suggest the use of this subscale—comparing it with the performance in the rest of the subscales—as an indicator to differentiate mild impairment from more severe states. More empirical evidence is needed to support the presence of this possible pattern of impairment, as well as to support the diagnostic utility of the memory scale in detecting mild cognitive impairment.

Regarding the results by subscale and the proposal of corrected versions, a detailed analysis is necessary.

Third, the location of the information curve peaks with respect to the continuum of trait level may be indicating a decline pattern, which begins with memory decline, then with language and orientation simultaneously, and finally ending with the visual recognition decline.

### Orientation

As for the Orientation scale, two of its items have malfunctioned. Although this could be explained from an occupational and/or cultural point of view, their exclusion does not diminish but rather increases the fit of the subscale. As for the item "date”, due to the participants’ demographic characteristics and age, it is possible that in most of their occupational activities there is no demand for the use of this information. As for "commune," since it is an administrative district, it requires some local administrative policy knowledge. Therefore, this item may have differential functioning due to educational levels. In both cases, not getting one of the items right could not necessarily be associated with a loss of orientation due to cognitive deterioration. We recommended not to consider these items in the total score of the subscale.

### Memory

We propose an alternative model by eliminating the items of the Working memory and Episodic coding tasks considering, on the one hand, the theoretical proposals [61–63] and, on the other, the results of the analysis. They showed a significant improvement of the global fit and a misfit was found in only one of its items.

According to the proposal that the tasks of episodic free recovery, declarative memory, and recognition correspond to indicators more suitable for memory function assessment [61–63], and to the possibility that the tasks of working memory and episodic coding could contribute to the lack of adjustment of the model, we have assessed a modified version of the memory subscale for our analyzes, composed only of the Episodic free recovery, Declarative memory, and Episodic recognition tasks.

As for the evocation of the three words (lemon-key-door), the characteristics of the task do not allow us to differentiate whether the person’s response depends on his or her working memory or on the evocation itself. On the contrary, on the items related to the repetition of name and address (Miguel González), the instructions involve sequential processes of repetition, similar to how other tests are widely used to evaluate episodic memory do [61]. From a statistical point of view, the model presents a significant improvement when the lemon-key-door items and the first item score of Miguel González are excluded. Nevertheless, we recommend maintaining the application of the repetition phase of Miguel González without scoring it, since it is required to assure the information codification process for its later evocation evaluation.

As we have previously mentioned, the values of the b-parameters indicate the point of the trait level at which the probability of hitting becomes greater than .5. Because the b-parameter is found in the trait continuum metric (theta), it is possible to determine the level of cognitive impairment in which people fail in their response [17–20].

Based on the values of the estimated b-parameters of the different tasks, the results show that the items of the Episodic free recovery task have greater sensitivity in mild levels of impairment (*b* = 0, mean of trail level), while items in the tasks of Declarative memory and Episodic recognition have greater sensitivity at more severe levels of deterioration (*b* = −1, one standard deviation below the mean). These findings support the possibility of establishing performance-based impairment levels against different memory tasks.

### Language

As for the language subscale, only 4 of the 25 items showed a lack of fit. In the case of the word repetition task, we recommend modifying its score by coding it in a dichotomous way (all correct words versus failing in at least one) since the intermediate score does not contribute to discrimination in evaluating the trait. We recommend that the remaining three items (rhinoceros, kangaroo and accordion) be eliminated or modified, given the possible cultural effect on their functioning.

### Visuospatial

The Visuospatial subscale showed an apparent global lack of fit. Given that the tasks of visoconstruction and recognition, although related, correspond to clearly differentiated tasks [69], we decided to specify both models separately. The results showed a lack of fit on the visoconstructive task, contrary to the recognition tasks, which showed an adequate fit. Developing studies that can improve the functioning of visoconstructive tasks and evaluate their contribution to visuospatial performance assessment is recommended.

There are some limitations to this study that are possible to highlight. Despite the use of a large sample of participants and that people with a diagnosis of dementia correspond to a similar percentage to the prevalence of dementia in the general population, it is possible that the small number of people with severe deterioration may partially affect the results. More studies are needed to consider a larger number of participants with severe levels of impairment. Given that this study only considered participants with a diagnosis of Alzheimer’s type dementia, subsequent studies should consider other types of dementias for the psychometric evaluation of the proposed version of the test under different conditions, and evaluate the possibility of detecting differential patterns of deterioration.

Although this study’s results are not conclusive, they present significant contributions to improving the overall functioning of the ACE-III. The findings presented can be beneficial for modifying and/or eliminating certain items from the subscales. Special attention is needed in the Attention subscale and the tasks of construction of the visuospatial subscale.

A special contribution are the findings regarding the supposed presence of a pattern of deterioration shown in the analysis of information function curves and the possibility of using the Memory subscale, especially the task of episodic free recovery, which can become an alternative for the detection of mild cognitive impairment. Additionally, obtaining scores by subscales can become a good alternative for elaborating deterioration profiles that can be transformed into indicators for the differential diagnosis for different types of courses of dementia. Studies of diagnostic utility are necessary to accumulate evidence to support this type of diagnostic use.

## Supporting information

S1 Table Parameters estimated and item fit of full version of orientation subscale. (DOCX)

S2 Table Parameters estimated and item fit of full version of memory subscale. (DOCX)

S3 Table Parameters estimated and item fit of full version of language subscale. (DOCX)

S4 Table Parameters estimated and item fit of full version of visual construction subscale. (DOCX)

S1 File (XLSX)
