# Supplementary material for: Psychometric properties of Addenbrooke’s Cognitive Examination III (ACE-III): An item response theory approach
Source: PLoS One. 2021 May 6;16(5):e0251137. doi: 10.1371/journal.pone.0251137 (PMC8101956; doi:10.1371/journal.pone.0251137)
Supplement: S1 Table — (DOCX) [file pone.0251137.s001.docx]

| **S 1. Table. Parameters estimated and item fit of full version of orientation subscale** | | | | | | | | | |
| --- | --- | --- | --- | --- | --- | --- | --- | --- | --- |
|  | **Parameters estimated** | | | | **Items fit indices** | | | |  |
|  | a | S.E. | b | S.E. | S-χ^2^ | df | p | RMSEA |  |
| **Day** | 2.609 | .266 | -1.481 | .082 | 8.402 | 6 | .210 | .019 |  |
| **Date** | 3.138 | .341 | -1.132 | .063 | 22.288 | 5 | .000 | .056 |  |
| **Month** | 4.747 | .638 | -1.307 | .062 | 6.838 | 5 | .233 | .018 |  |
| **Year** | 3.891 | .483 | -.993 | .055 | 2.299 | 4 | .681 | .000 |  |
| **Season** | 2.295 | .224 | -1.237 | .076 | 8.902 | 6 | .179 | .021 |  |
| **Country** | 2.715 | .411 | -2.420 | .157 | 8.633 | 4 | .071 | .032 |  |
| **City** | 3.950 | .645 | -2.034 | .103 | 4.470 | 3 | .215 | .021 |  |
| **Commune** | 1.140 | .119 | -.943 | .097 | 21.738 | 6 | .001 | .048 |  |
| **Street** | 3.224 | .373 | -1.651 | .084 | 5.955 | 6 | .428 | .000 |  |
| **Number** | 1.831 | .193 | -1.838 | .123 | 2.138 | 7 | .952 | .000 |  |
| Note: a = a-parameter; S.E. = Standard error; b = b-parameter; S-χ^2^ = Goodness of fit index S-χ^2^; df = degrees of freedom; p = p-value; RMSEA = Root mean square error of approximation. | | | | | | | | |  |
