# Supplementary material for: Psychometric properties of Addenbrooke’s Cognitive Examination III (ACE-III): An item response theory approach
Source: PLoS One. 2021 May 6;16(5):e0251137. doi: 10.1371/journal.pone.0251137 (PMC8101956; doi:10.1371/journal.pone.0251137)
Supplement: S2 Table — (DOCX) [file pone.0251137.s002.docx]

| **S 2. Table. Parameters estimated and item fit of full version of memory subscale** | | | | | | | | | | | |
| --- | --- | --- | --- | --- | --- | --- | --- | --- | --- | --- | --- |
|  |  | **Parameters estimated** | | | | **Items fit indices** | | | |  |  |
|  |  | **a** | **S.E.** | **b** | **S.E.** | **S-χ^2^** | **df** | **p** | **RMSEA** |  |  |
| **Working memory** | **Lemon** | 1.864 | .158 | -1.435 | .089 | 38.379 | 20 | .008 | .029 |  |  |
|  | **Key** | 1.342 | .110 | -.959 | .083 | 29.320 | 21 | .107 | .019 |  |  |
|  | **Door** | 1.303 | .107 | -.911 | .082 | 20.256 | 21 | .505 | .000 |  |  |
| **Episodic coding** | **Miguel (1)** | 1.995 | .189 | -1.792 | .108 | 13.364 | 19 | .819 | .000 |  |  |
|  | **González (1)** | 2.096 | .200 | -1.787 | .105 | 14.934 | 19 | .727 | .000 |  |  |
|  | **Avenida (1)** | 1.650 | .141 | -1.449 | .096 | 35.581 | 21 | .024 | .025 |  |  |
|  | **Imperial (1)** | 2.208 | .169 | -.870 | .060 | 23.711 | 20 | .255 | .013 |  |  |
|  | **68 (1)** | 2.042 | .157 | -.923 | .064 | 31.541 | 20 | .048 | .023 |  |  |
|  | **Caldera (1)** | 1.646 | .137 | -1.352 | .091 | 24.180 | 21 | .284 | .012 |  |  |
|  | **Copiapó (1)** | 2.063 | .186 | -1.600 | .094 | 14.858 | 20 | .785 | .000 |  |  |
| **Declarative memory** | **Actual president** | 2.485 | .216 | -1.367 | .075 | 25.787 | 18 | .105 | .020 |  |  |
|  | **Military government** | 2.518 | .225 | -1.429 | .078 | 23.110 | 18 | .186 | .016 |  |  |
|  | **USA president** | 1.552 | .118 | -.309 | .058 | 12.122 | 20 | .912 | .000 |  |  |
|  | **Murdered USA pres.** | 1.993 | .152 | -.875 | .063 | 12.869 | 20 | .883 | .000 |  |  |
| **Episodic Free recovery** | **Miguel (2)** | 1.614 | .125 | .279 | .055 | 14.619 | 18 | .688 | .000 |  |  |
|  | **González (2)** | 2.255 | .166 | -.023 | .046 | 14.408 | 16 | .568 | .000 |  |  |
|  | **Avenida (2)** | 2.083 | .154 | .047 | .048 | 18.648 | 17 | .349 | .009 |  |  |
|  | **Imperial (2)** | 2.153 | .170 | .475 | .051 | 19.341 | 13 | .113 | .021 |  |  |
|  | **68 (2)** | 3.061 | .239 | .005 | .042 | 8.713 | 13 | .794 | .000 |  |  |
|  | **Caldera (2)** | 2.515 | .190 | .059 | .045 | 22.343 | 15 | .099 | .021 |  |  |
|  | **Copiapó (2)** | 3.109 | .239 | -.443 | .045 | 12.469 | 15 | .643 | .000 |  |  |
| **Episodic Recognition** | **Miguel González (3)** | 2.016 | .153 | -.866 | .063 | 31.732 | 20 | .046 | .023 |  |  |
|  | **Avenida Imperial (3)** | 1.931 | .149 | -.970 | .068 | 35.532 | 20 | .017 | .026 |  |  |
|  | **68 (3)** | 2.001 | .153 | -.877 | .063 | 29.038 | 20 | .087 | .020 |  |  |
|  | **Caldera (3)** | 2.489 | .191 | -.817 | .056 | 32.549 | 18 | .019 | .027 |  |  |
|  | **Copiapó (3)** | 3.314 | .313 | -1.270 | .064 | 11.170 | 16 | .799 | .000 |  |  |
| Note: a = a-parameter; S.E. = Standard error; b = b-parameter; S-χ^2^ = Goodness of fit index S-χ^2^; df = degrees of freedom; p = p-value; RMSEA = Root mean square error of approximation. | | | | | | | | | | |  |
