# Supplementary material for: Psychometric properties of Addenbrooke’s Cognitive Examination III (ACE-III): An item response theory approach
Source: PLoS One. 2021 May 6;16(5):e0251137. doi: 10.1371/journal.pone.0251137 (PMC8101956; doi:10.1371/journal.pone.0251137)
Supplement: S3 Table — (DOCX) [file pone.0251137.s003.docx]

| **S 3. Table. Parameters estimated and item fit of full version of language subscale** | | | | | | | | |
| --- | --- | --- | --- | --- | --- | --- | --- | --- |
|  | **Parameters estimated** | | | | **Items fit indices** | | | |
|  | **a** | **S.E.** | **b** | **S.E.** | **S-χ^2^** | **df** | **p** | **RMSEA** |
| **Instruction 1** | 1.948 | .183 | -1.701 | .105 | 21.751 | 20 | .354 | .009 |
| **Instruction 2** | 2.014 | .209 | -2.025 | .127 | 14.802 | 21 | .833 | .000 |
| **Instruction 3** | 1.230 | .111 | -1.264 | .105 | 22.564 | 20 | .311 | .011 |
| **Repeat phrase 1** | 1.386 | .120 | -1.154 | .091 | 26.609 | 20 | .147 | .017 |
| **Repeat phrase 2** | 1.792 | .158 | -1.447 | .093 | 23.454 | 20 | .267 | .012 |
| **Create phrase 1** | 2.631 | .240 | -1.378 | .075 | 15.084 | 17 | .589 | .000 |
| **Create phrase 2** | 1.876 | .150 | -.612 | .058 | 5.058 | 16 | .995 | .000 |
| **Repeat words 1** | 1.769 | .147 | -1.163 | .079 | 50.049 | 18 | .000 | .039 |
| **Repeat words 2** | 1.534 | .125 | -.063 | .055 | 13.795 | 15 | .541 | .000 |
| **Spoon** | 4.350 | .921 | -2.433 | .130 | 4.366 | 3 | .225 | .020 |
| **Book** | 1.955 | .200 | -1.992 | .126 | 23.156 | 21 | .336 | .009 |
| **Kangaroo** | 2.231 | .189 | -1.119 | .069 | 27.375 | 18 | .072 | .021 |
| **Penguin** | 3.007 | .290 | -1.418 | .073 | 19.884 | 16 | .225 | .015 |
| **Anchor** | 2.344 | .210 | -1.402 | .079 | 6.574 | 18 | .993 | .000 |
| **Camel** | 2.302 | .210 | -1.486 | .084 | 24.594 | 18 | .137 | .018 |
| **Harp** | 2.543 | .219 | -1.146 | .066 | 21.398 | 17 | .209 | .015 |
| **Rhino** | 1.348 | .113 | -.297 | .062 | 11.741 | 17 | .816 | .000 |
| **Barrel** | 1.326 | .123 | -1.526 | .117 | 14.462 | 20 | .806 | .000 |
| **Crown** | 3.495 | .368 | -1.494 | .072 | 17.769 | 15 | .275 | .013 |
| **Crocodile** | 1.852 | .156 | -1.200 | .079 | 15.284 | 18 | .642 | .000 |
| **Accordion** | 1.856 | .172 | -1.656 | .104 | 44.46 | 20 | .001 | .033 |
| **Monarchy** | 2.773 | .261 | -1.440 | .076 | 18.357 | 17 | .367 | .008 |
| **Reptile** | 2.878 | .282 | -1.529 | .079 | 9.207 | 17 | .934 | .000 |
| **Antarctica** | 3.344 | .335 | -1.397 | .069 | 8.102 | 15 | .920 | .000 |
| **Nautical** | 2.370 | .205 | -1.234 | .071 | 20.561 | 18 | .302 | .011 |
| **Repeat 4 words** | 1.637 | .134 | .104 | .053 | 16.842 | 13 | .207 | .016 |
| Note: a = a-parameter; S.E. = Standard error; b = b-parameter; S-χ2 = Goodness of fit index S-χ2; df = degrees of freedom; p = p-value; RMSEA = Root mean square error of approximation. | | | | | | | | |
