# Supplementary material for: Psychometric properties of Addenbrooke’s Cognitive Examination III (ACE-III): An item response theory approach
Source: PLoS One. 2021 May 6;16(5):e0251137. doi: 10.1371/journal.pone.0251137 (PMC8101956; doi:10.1371/journal.pone.0251137)
Supplement: S4 Table — (DOCX) [file pone.0251137.s004.docx]

| **S 4. Table. Parameters estimated and item fit of full version of visual construction subscale** | | | | | | | | | |
| --- | --- | --- | --- | --- | --- | --- | --- | --- | --- |
|  | **Parameters estimated** | | | | **Items fit indices** | | | | |
|  | **a** | **S.E.** | **b** | **S.E.** | **S-χ^2^** | **df** | **p** | **RMSEA** |  |
| **Infinity** | 2.044 | .174 | -.559 | .055 | 25.956 | 8 | .001 | .045 |  |
| **Cube 1** | 2.370 | .208 | -.690 | .055 | 24.205 | 8 | .002 | .042 |  |
| **Cube 2** | 1.746 | .154 | -.051 | .052 | 11.545 | 7 | .117 | .024 |  |
| **Circle** | 2.910 | .316 | -1.679 | .092 | 21.163 | 9 | .012 | .035 |  |
| **Number 1** | 3.773 | .534 | -.701 | .050 | 34.213 | 7 | .000 | .059 |  |
| **Number 2** | 3.899 | .580 | -.548 | .046 | 23.386 | 6 | .001 | .051 |  |
| **Clock hands 1** | 3.484 | .340 | -.618 | .047 | 9.306 | 7 | .231 | .017 |  |
| **Clock hand 2** | 2.613 | .244 | -.103 | .044 | 13.809 | 6 | .032 | .034 |  |
| **8 point** | 2.422 | .267 | -1.989 | .119 | 10.444 | 11 | .491 | .000 |  |
| **10 point** | 1.714 | .170 | -1.881 | .129 | 21.330 | 12 | .046 | .026 |  |
| **7 point** | 1.545 | .163 | -2.172 | .162 | 23.912 | 12 | .021 | .030 |  |
| **9 point** | 1.951 | .196 | -1.872 | .121 | 11.660 | 12 | .473 | .000 |  |
| **Letter K** | 1.676 | .164 | -1.813 | .126 | 7.038 | 12 | .855 | .000 |  |
| **Letter M** | 3.546 | .490 | -1.967 | .105 | 4.798 | 5 | .441 | .000 |  |
| **Letter A** | 3.347 | .484 | -2.190 | .121 | 9.039 | 5 | .108 | .027 |  |
| **Letter T** | 3.455 | .520 | -2.235 | .123 | 6.428 | 5 | .267 | .016 |  |
| Note: a = a-parameter; S.E. = Standard error; b = b-parameter; S-χ2 = Goodness of fit index S-χ2; df = degrees of freedom; p = p-value; RMSEA = Root mean square error of approximation. | | | | | | | | |  |
